# Sepsis secondary to postoperative *Enterococcus gallinarum* infection in a patient with rectal cancer: A case report

**DOI:** 10.1097/MD.0000000000046454

**Published:** 2026-05-12

**Authors:** Xiaoyu Zhang, Lan Li, Ruifeng Huang

**Affiliations:** a The First School of Clinical Medicine, Guizhou University of Traditional Chinese Medicine, Guiyang, China; b Department of Critical Care Medicine, The First Affiliated Hospital of Guizhou University of Traditional Chinese Medicine, Guiyang, China.

**Keywords:** antibiotics, *Enterococcus gallinarum*, etiological culture, sepsis

## Abstract

**Rationale:**

*Enterococcus gallinarum*, while of relatively low virulence, is an emerging multidrug-resistant opportunistic pathogen. This case report aims to highlight the diagnostic and therapeutic challenges posed by this organism, particularly in immunocompromised hosts following major surgery, and to emphasize the importance of early microbiological identification and targeted therapy in such scenarios.

**Patient concerns:**

A 55-year-old woman was admitted to our hospital with a 1-month history of recurrent abdominal pain and bloating. She underwent surgical intervention and subsequently developed septic shock. Despite empiric therapy with imipenem–cilastatin (1000 mg every 8 hours) plus vancomycin (1000 mg every 12 hours), her condition deteriorated, presenting with persistent fever and progressive multiorgan dysfunction syndrome.

**Diagnoses:**

*Enterococcus gallinarum* (Group D), intrinsically resistant to vancomycin, was isolated from the wound secretion culture.

**Interventions:**

According to the culture results, antibiotic therapy was adjusted to levofloxacin (300 mg once daily) plus meropenem (1000 mg every 8 hours).

**Outcomes:**

After the adjustment of antibiotics, the patient’s body temperature normalized within days. Following 1 week of targeted antibiotic therapy, all infection markers (including white blood cell count and procalcitonin) normalized. The antibiotic regimen was successfully de-escalated, and the patient was discharged after full clinical recovery.

**Lessons:**

The diagnosis and management of sepsis due to postoperative *E gallinarum* infection should focus on preventing and controlling postoperative complications, performing early etiological cultures, and selecting appropriate antibiotics.

## 1. Introduction

*Enterococcus gallinarum* is a facultative anaerobic, catalase-negative, Gram-positive Group D coccus that usually forms chains.^[1,2]^ It is also commonly found in the normal intestinal microbiota of birds and humans.^[3]^ As an opportunistic pathogen, *E gallinarum* is primarily associated with nosocomial infections. However, its prevalence is relatively low. In 1 study of 403 *Enterococcus* isolates, only 11 were identified as *E gallinarum*.^[4]^ Another study reported that *E gallinarum* accounted for 4.8% of *Enterococcus* infections, which remain uncommon in clinical practice and are mainly described in case reports.^[5]^ Here, a case of sepsis caused by *E gallinarum* following rectal cancer surgery is presented, aiming to highlight the clinical diagnosis and management of this rare condition.

## 2. Case presentation

A 55-year-old woman was admitted to our hospital with a 1-month history of recurrent abdominal pain and bloating. The abdominal pain was colicky and was accompanied by nausea, vomiting, and constipation. Three months earlier, she had been diagnosed with rectal cancer at another hospital, where she presented with perianal pain and hematochezia and subsequently underwent surgical resection.

**Figure 1.**
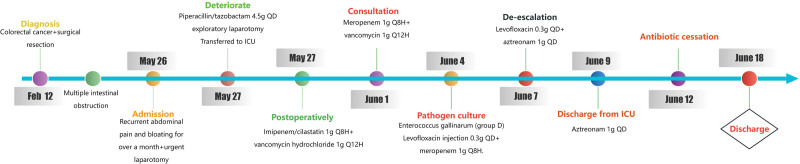
Antibiotic therapy of the patient over the course of admission.

The patient had multiple admissions to the medical facility for postoperative intestinal obstruction and received conservative management. Her medical history was significant for cholelithiasis treated with cholecystectomy, and no other major comorbidities were noted. After admission, hypotension, electrolyte imbalance, and fever rapidly developed. Due to ineffective conservative management, an urgent laparotomy was performed. Blood cultures were collected, and empiric antibiotic therapy was initiated before surgery. During surgery, wound secretion cultures were also obtained, and the patient was transferred to the intensive care unit for postoperative monitoring and treatment (Fig. 1). Upon intensive care unit admission, her vital signs were as follows: temperature was 37.9°C, blood pressure was 75/53 mm Hg (supported by phenylephrine infusion), heart rate was 140 beats per minute, respiratory rate was 16 breaths per minute, and oxygen saturation was 99% with endotracheal intubation and mechanical ventilation. Chest auscultation revealed coarse breath sounds bilaterally, accompanied by dry and wet rales. Abdominal examination showed a soft, tympanitic abdomen, but tenderness, rebound tenderness, and muscle tone could not be assessed due to patient noncooperation. No bowel movements or flatus were noted after surgery.

**Figure 2.**
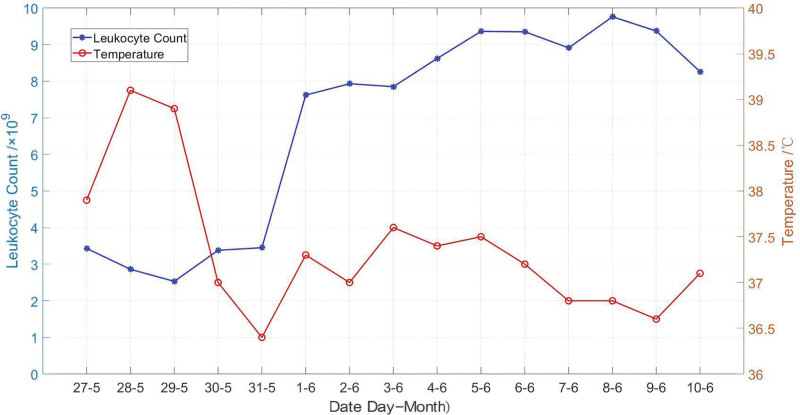
Fluctuations in body temperature and white blood cell count from May 27 to June 10, 2024.

Laboratory tests revealed a white blood cell count of 3.43 × 10^9^/L (reference range 3.5–9.5 × 10^9^/L) (Fig. 2), a neutrophil percentage of 79.7%, and a procalcitonin level of 65.72 ng/L. A severe intra-abdominal infection was suspected, and empiric antimicrobial therapy with imipenem–cilastatin (1000 mg every 8 hours) plus vancomycin (1000 mg every 12 hours) was administered. During this period, the patient developed multiple organ dysfunction syndrome involving the circulatory, hematologic, renal, and hepatic systems. Despite continuous renal replacement therapy, fluid resuscitation, vasoactive medications, blood transfusions, and other interventions, her condition did not improve significantly. After multidisciplinary consultation, the antibiotic regimen was changed to meropenem (1000 mg every 8 hours) plus vancomycin (1000 mg every 12 hours). Concurrently, blood cultures yielded no bacterial growth. On postoperative day 8, *E gallinarum* (Group D) was isolated from cultured wound secretions. The isolated strain exhibited intrinsic resistance to vancomycin, quinupristin/dalfopristin, trimethoprim, penicillin G, and ampicillin, but remained susceptible to high-dose gentamicin, high-dose streptomycin, levofloxacin, ciprofloxacin, erythromycin, linezolid, tetracycline, and tigecycline. Antibiotic therapy was consequently adjusted based on sensitivity results to levofloxacin (300 mg once daily) combined with meropenem (1000 mg every 8 hours). After initiation of this regimen, the patient’s temperature returned to normal, indicating successful infection management. Subsequently, antibiotic therapy was de-escalated to levofloxacin and aztreonam. Following 1 week of antimicrobial treatment, all infection markers normalized, and the patient was transferred to a specialized department for continuation of aztreonam therapy. Antibiotics were eventually discontinued, and the patient was discharged upon clinical improvement.

## 3. Discussion

The patient described here was immunocompromised and underwent invasive surgery, eventually progressing to sepsis and multiple organ dysfunction. This case provides valuable clinical insights into the management of *E gallinarum* infections. Clinicians should remain vigilant not only for common bacterial pathogens but also for uncommon organisms, particularly in patients who undergo invasive surgical procedures.

Postoperative infections remain major complications following colorectal cancer (CRC) surgery, with reported incidence ranging from 10% to 30%, depending on patient comorbidities, surgical techniques, and perioperative management practices.^[6]^ The most frequent complications include surgical site infections, intra-abdominal abscess formation, and bloodstream infections, significantly increasing morbidity, hospitalization duration, and healthcare costs.^[7]^ The systemic inflammatory response associated with cancer is a crucial indicator of tumor progression. Accumulating evidence suggests that the neutrophil–lymphocyte ratio is a reliable biomarker for prognosis in various malignancies. In patients with stage I–III CRC who underwent curative surgical resection, an neutrophil–lymphocyte ratio >2.72 was independently predictive of overall survival.^[8]^ Low serum calcium levels have also been associated with postoperative outcomes in CRC patients.^[9]^ Additionally, the prognostic significance of combining platelet-albumin ratios and cancer inflammation index, and early prediction of anastomotic leakage via nutritional and inflammation-related biomarkers, further emphasize the clinical relevance of patients’ immune-inflammatory and nutritional status.^[10,11]^

Gram-negative bacteria, particularly *Escherichia coli* and *Klebsiella pneumoniae*, along with *Enterococcus* species, are frequently implicated in postoperative infections after CRC surgery. Among *Enterococcus* species, vancomycin-resistant enterococci present a significant clinical challenge due to limited therapeutic options. In this context, our case underscores *E gallinarum* as an uncommon yet clinically important pathogen capable of causing severe postoperative sepsis in CRC patients. Although *E gallinarum* is normally a commensal organism within the human gut microbiota, it possesses pathogenic potential, causing severe infections under specific clinical conditions. Documented *E gallinarum*-associated infections are diverse, encompassing infective endocarditis, meningitis, peritonitis, cholangitis, and bacteremia, particularly among immunocompromised patients or those undergoing invasive medical procedures.^[12–15]^ Although relatively uncommon, these infections are often severe and associated with significant morbidity. The increasing detection of *E gallinarum* in infections involving multiple organ systems underscores the importance of considering this pathogen in the differential diagnosis, especially when patients do not respond to conventional empirical antibiotic therapy.

*Enterococcus gallinarum* is a multidrug-resistant bacterium with increasing rates of clinical infection and isolation. A previous study in 2011 reported 102 strains isolated from 15 hospitals across China, with an isolation rate of 2.2% and a vancomycin resistance rate of 82.7%.^[16]^ It ranks third among enterococcal bloodstream infections caused by vancomycin-resistant strains. *Enterococcus gallinarum* harbors intrinsic resistance to vancomycin due to the presence of the vanC gene, encoding enzymes that modify the termini of peptidoglycan precursors. Instead of synthesizing the canonical D-alanine–D-alanine, these enzymes produce low-affinity ligands such as D-alanine–D-serine or D-alanine–D-lactate. These modified precursors replace the original D-alanine–D-alanine at the terminal ends of peptidoglycan chains, disrupting hydrogen bonding between vancomycin and its target sites. Consequently, vancomycin is sterically hindered from binding to bacterial cell wall precursors, thus conferring resistance.^[17]^ Furthermore, *E gallinarum* can evolve within the host, differentiating into distinct subpopulations. Certain subpopulations may translocate across the intestinal barrier, persistently colonize extraintestinal organs, and induce chronic inflammation and associated pathologies, thereby increasing their pathogenicity.^[18]^ Additionally, *E gallinarum* expresses pili that play a critical role in host adhesion. Notably, similar to other Enterococcus species, it exhibits the ability to form biofilms, enabling colonization on catheters and other surfaces. Biofilm-embedded bacteria are notoriously difficult to eradicate due to their enhanced resistance to phagocytosis and antimicrobial agents.^[15]^

Our patient was a poultry farmer with a history of bird exposure, raising the possibility of an *Enterococcus* infection originating from quails. Moreover, the patient’s transfer among multiple healthcare institutions during her diagnostic and therapeutic process may have significantly increased her risk for nosocomial acquisition of this pathogen. It is essential to obtain and submit appropriate specimens for culture before initiating antimicrobial therapy. In the present case, discrepancies were observed in the patient’s culture results, suggesting that in septic patients, negative blood cultures do not necessarily exclude bacterial infection. Such discrepancies may reflect differences in infection sources or the patient’s immune status. Furthermore, the patient’s multiple courses of antibiotics for postoperative intestinal obstruction likely contributed to immune compromise, increasing susceptibility to opportunistic infections. These findings highlight the need for clinicians to interpret blood culture results with caution and to corroborate these results with additional cultures to more accurately identify the infectious source.

Unfortunately, this study has several limitations inherent to its design and context. First, as a single case report, its findings and conclusions may not be generalizable to broader patient populations. Second, the diagnostic process was hampered by the limited availability and high cost of metagenomic next-generation sequencing at our institution at the time, which precluded its use. The unavailability of this advanced diagnostic tool likely contributed to a delay in identifying *E gallinarum* and subsequently adjusting to targeted therapy in a more timely manner.^[19]^ Third, the patient’s complex transfer history among multiple medical institutions may have introduced confounding factors, making it challenging to trace the exact source and timeline of the infection with certainty. These limitations should be considered when interpreting the findings of this report.

In summary, although clinically rare, *E gallinarum* should be considered a potential pathogen in immunocompromised patients undergoing invasive surgery. Thorough medical history assessment, tracing of potential transmission routes, prompt etiological testing, and early administration of effective antibiotics are critical for managing *E gallinarum* infections.

## Acknowledgments

We thank the efforts and contributions of the reported patients and all the clinical staff in this study.

## Author contributions

**Conceptualization**: Xiaoyu Zhang, Ruifeng Huang.

**Data curation**: Xiaoyu Zhang, Lan Li, Ruifeng Huang.

**Formal analysis**: Lan Li, Ruifeng Huang.

**Writing – original draft**: Xiaoyu Zhang.

**Writing – review & editing**: Xiaoyu Zhang, Lan Li, Ruifeng Huang.
